# Offline low-frequency rTMS of the primary and premotor cortices does not impact motor sequence memory consolidation despite modulation of corticospinal excitability

**DOI:** 10.1038/s41598-021-03737-3

**Published:** 2021-12-17

**Authors:** Felix Psurek, Bradley Ross King, Joseph Classen, Jost-Julian Rumpf

**Affiliations:** 1 Department of Neurology, University of Leipzig, Liebigstr. 20, 04103 Leipzig, Germany; 2 Department of Health & Kinesiology, University of Utah, Utah, USA

**Keywords:** Synaptic plasticity, Motor control, Motor cortex, Premotor cortex, Learning and memory, Consolidation

## Abstract

Motor skills are acquired and refined across alternating phases of practice (online) and subsequent consolidation in the absence of further skill execution (offline). Both stages of learning are sustained by dynamic interactions within a widespread motor learning network including the premotor and primary motor cortices. Here, we aimed to investigate the role of the dorsal premotor cortex (dPMC) and its interaction with the primary motor cortex (M1) during motor memory consolidation. Forty-eight healthy human participants (age 22.1 ± 3.1 years) were assigned to three different groups corresponding to either low-frequency (1 Hz) repetitive transcranial magnetic stimulation (rTMS) of left dPMC, rTMS of left M1, or sham rTMS. rTMS was applied immediately after explicit motor sequence training with the right hand. Motor evoked potentials were recorded before training and after rTMS to assess potential stimulation-induced changes in corticospinal excitability (CSE). Participants were retested on motor sequence performance after eight hours to assess consolidation. While rTMS of dPMC significantly increased CSE and rTMS of M1 significantly decreased CSE, no CSE modulation was induced by sham rTMS. However, all groups demonstrated similar significant offline learning indicating that consolidation was not modulated by the post-training low-frequency rTMS intervention despite evidence of an interaction of dPMC and M1 at the level of CSE. Motor memory consolidation ensuing explicit motor sequence training seems to be a rather robust process that is not affected by low-frequency rTMS-induced perturbations of dPMC or M1. Findings further indicate that consolidation of explicitly acquired motor skills is neither mediated nor reflected by post-training CSE.

## Introduction

The acquisition of a new motor skill is a multi-staged process that evolves both “online”, concurrent with repeated skill execution, and “offline”, between training sessions^1,2^. The latter is referred to as motor memory consolidation during which the initially fragile training-induced internal skill model is secured against interference and transformed into a more robust representation in the absence of further skill execution^1,3–6^. In recent years, a large body of evidence has pointed to a fundamental role of the primary motor cortex (M1) for processing offline motor memory consolidation^2–4,7–9^. With respect to the neurophysiological mechanisms that may underlie the role of M1 in motor sequence consolidation, Tunovic and co-workers^8^ reported that the induction and magnitude of offline-performance increments following motor sequence training was associated with the level of post-training corticospinal excitability (CSE). However, a large body of evidence shows that sequential motor skill acquisition is sustained by specific and temporally dynamic interactions between multiple nodes of a widespread neural network that—in addition to M1—encompasses secondary motor cortical areas, parietal areas, as well as basal ganglia, hippocampus, the cerebellum, and the spinal cord^9–15^. Within this network, recent research has identified the dorsal premotor cortex (dPMC) as a region that (i) has been shown to modulate excitability and plasticity in M1 by interconnections^16–19^, (ii) plays an important role in the process of online motor memory formation^9,20–22^, and (iii) may thus also be crucially involved in offline motor memory consolidation after motor practice. The relevant involvement of dPMC in offline motor memory consolidation is supported by several studies demonstrating modulation of offline motor sequence consolidation by non-invasive brain stimulation (NIBS) of the dPMC^23–28^. However, the interpretation of these previous findings in terms of a crucial role of the dPMC specifically during post-training motor memory consolidation is difficult, as behavioural effects induced by “excitatory” or “inhibitory” NIBS protocols were inconsistent and the timing of the NIBS intervention in relation to the motor training session (i.e., pre, during, or after) differed across studies.

In the current study, we investigated dPMC and M1 specifically in terms of their role in post-training offline motor memory processing. Findings of previous studies that investigated implicit motor sequence learning suggest that immediate post-training “inhibitory” low-frequency rTMS of the dPMC promotes consolidation^24^ while post-training low-frequency rTMS-induced treatment of M1 blocks motor memory consolidation^4^. This may point towards opposing roles of the dPMC and M1 during offline motor memory processing after implicit skill acquisition. However, given the evidence that the premotor cortex is particularly critical in explicit acquisition of sequential motor skills^22,29–32^, we applied a purely explicit and ecologically valid motor sequence learning paradigm. Twenty minutes of offline (i.e., post-training) low-frequency repetitive transcranial magnetic stimulation (rTMS) was used to transiently disrupt motor memory processing in the dPMC and M1 during the early consolidation process immediately after a motor sequence training session in the morning. Effects of post-training stimulation on behavioral markers of consolidation were assessed with a delayed retest in the afternoon administered eight hours after the post-training rTMS intervention. Additionally, we assessed post-training rTMS-induced changes in CSE to gain insight into the potential interaction of the dPMC with M1 during early motor memory consolidation following explicit skill acquisition and to further explore the association of post-training CSE changes with offline consolidation. If malleability of motor memory consolidation by offline NIBS of the dPMC and M1 proves to be a robust finding, it would be conceivable to explore its potential as a therapeutic tool to specifically target and modulate motor memory consolidation in the future.

## Methods

### Ethical standards

The study protocol was approved by the institutional ethical standards committee at the University of Leipzig (registration code: 326/18-ek). All methods were performed in accordance with the relevant guidelines and regulations and all participants provided written informed consent before study-related procedures were conducted.

### Participants

Fifty-two young, healthy, and right-handed participants aged between 18 and 30 years (39 female; mean age 22.1 ± 3.1 years) were recruited via social media or adverts on the local notice board at the University of Leipzig and completed the experiment. All participants were naïve to the motor sequence learning task and the purpose of the experiment. None of the participants had previously experienced 1 Hz rTMS or any other type of transcranial magnetic stimulation. Right-handedness was verified using the Edinburgh-Handedness-Inventory (EHI^33^). None of the participants reported a history of neurological or psychiatric disorders (including abuse of alcohol or other illicit drugs) and none of the participants had a relevant medical condition (e.g., rheumatoid arthritis) that might impair task execution. Exclusion criteria further encompassed having been trained as a professional typist or a professional musician to exclude individuals in which dexterous sequential finger movements like in the task employed in our study were likely “overtrained”, which might confound the rate and magnitude of online and offline task learning. Additional TMS-specific exclusion criteria comprised a history of seizure, central nervous system active medication, and current pregnancy. All participants were further screened for symptoms of depression using the short version of the Beck-Depression-Inventory^34^. The Stanford-Sleepiness-Scale^35^ was applied before the initial training session in the morning and the delayed retest session in the evening to assess the possibility that the task execution was confounded by relevant between-group differences in terms of sleepiness/vigilance. Data of four participants were excluded due to insufficient learning of the task as indicated by a negative learning slope across the initial training session (3 participants in the M1 rTMS group, 1 participant in the dPMC rTMS group). The final data set, therefore, comprised experimental data of 48 participants (demographic information is detailed in Table 1).

**Table 1:** Group characteristics.

Group	N	Age, years	Sex, w/m	SSS_training	SSS_retest	BDI, score	EHI, score	RMT, % stimulator output	MEPpre, % stimulator output
M1 rTMS	16	23.2 ± 3.4	13/3	1.81 ± 0.75	2.50 ± 1.27	3.6 ± 2.9	78.5 ± 17.2	33.1 ± 4.1	39.9 ± 4.4
dPMC rTMS	16	20.9 ± 2.2	12/4	2.19 ± 0.54	2.81 ± 0.83	2.4 ± 1.5	80.5 ± 23.2	33.4 ± 4.0	40.5 ± 4.4
SHAM rTMS	16	22.1 ± 3.4	11/5	2.25 ± 0A.58	2.31 ± 0.79	2.3 ± 3.0	82.8 ± 15.4	35.8 ± 5.3	41.2 ± 6.1

Stanford-Sleepiness-Scale score before training (SSS_training) and before retest (SSS_retest); *N* number of participants, *BDI* Beck-Depression-Inventory, *EHI* Edinburgh-Handedness-Inventory, *RMT* Resting Motor Threshold, *MEPpre* individual stimulation intensity to elicit MEPs of ~ 1 mV at baseline. All values (excluding sex and number of participants) are displayed as mean ± standard deviation.

### Experimental procedure

**Figure 1:**
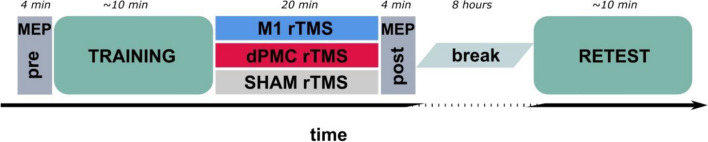
Experimental design. At the onset of the experiment, 20 motor evoked potentials (MEPs) of the right abductor pollicis brevis muscle (APB) were evoked at 0.1 Hz with the stimulator output set to an individually adjusted intensity to elicit MEPs of ~ 1 mV (MEPpre). This baseline assessment of corticospinal excitability (CSE) was followed by 14 blocks of task training with the right hand (TRAINING). Immediately after termination of TRAINING, participants received low-frequency (1 Hz) repetitive transcranial magnetic stimulation (rTMS) of either the left primary motor cortex (M1 rTMS), the dorsal premotor cortex (dPMC rTMS), or SHAM rTMS. 20 MEPs from the APB were again recorded (MEPpost) with the same individually adjusted stimulator output level as during the MEPpre assessment. Participants were retested on task performance (14 blocks) after an interval of 8 h (RETEST).

The experiment consisted of a motor sequence training session in the morning and a delayed retest session after eight hours to assess offline performance changes. Participants were randomly assigned to one of three different groups corresponding to three different post-training rTMS interventions (i.e., rTMS applied to the primary motor cortex, M1 group; rTMS applied to the dorsal premotor cortex, dPMC group; sham rTMS, SHAM rTMS group). Immediately following the completion of the initial training session post-training 1 Hz rTMS was applied for 20 min using individualized stimulation intensities (for details see below). CSE was assessed by recording TMS-induced motor-evoked potentials from the right abductor pollicis brevis muscle (APB) before the initial training session (MEPpre) and immediately after termination of the post-training rTMS intervention (MEPpost; Fig. 1).

### Motor sequence learning task

To assess explicit motor sequence learning, we applied a modified version of the explicit sequential finger-tapping task introduced by Karni and colleagues^7^. Participants were asked to perform a five-element finger-tapping sequence (4-1-3-2-4; where 1 = index finger, 2 = middle finger, 3 = ring finger, 4 = little finger) on a four-button keyboard with the right hand. Prior to the beginning of the actual training session, participants were required to correctly reproduce the finger-tapping sequence on the keyboard three times in a row to verify explicit knowledge of the sequence. Both the training session and the delayed retest session after eight hours encompassed 14 blocks of successive sequence execution, which were separated by 25-s rest intervals. A green visual cue displayed on a computer monitor in front of the participants indicated an active training block, while a rest period was indicated by a switch of the cue colour to red (i.e., after 60 key taps). Participants were instructed to perform the finger-tapping sequence as fast as possible, while making as few errors as possible. Unbeknownst to the participants, each block of active task execution was terminated after 60 key taps, resulting in a maximum number of 12 correct sequences per block. This design ensures that all participants received the same amount of training (i.e., performed the same number of finger movements).

### Transcranial magnetic stimulation

Transcranial magnetic stimulation was applied by a MagPro X100 (MagVenture, Farum, Denmark) connected to a MagVenture MC-B70 70 mm figure‐of‐eight coil. The stimulation coil was placed tangentially over the left hemisphere and the handle of the coil was pointed diagonally to the floor behind the subject with an angle of approximately 45 degrees. Electromyography of the right abductor pollicis brevis muscle (APB) was recorded using a Digitimer D360 (Digitimer Ltd., Letchworth Garden, UK). The “motor hotspot” for stimulation of the M1 hand area was identified by applying low-frequency (< 0.2 Hz) stimulation at multiple sites likely overlying left M1 while recording surface EMG from the right APB. We then obtained individual APB resting motor thresholds (RMT) using threshold hunting^36^. BrainSight 2 Neuronavigation (Brain Products, Gilching, Germany) aided to guide constant coil positioning throughout the experiment via virtual landmarks on the scalp. The post-training rTMS intervention encompassed the application of 1200 TMS pulses at a frequency of 1 Hz (i.e., 20 min of stimulation) with the stimulator output intensity set to 110% of the individual RMT. For the post-training rTMS intervention in the M1 group, the coil was placed over the individual “motor hotspot”, whereas post-training rTMS of the dorsal premotor cortex in the dPMC group was applied with the similarly oriented coil placed 2.5 cm anterior of the APB “motor hotspot”^17,18^. In the SHAM group, rTMS was applied at the same frequency and stimulator output intensity, but the coil was placed vertically to the scalp approximately over M1. This procedure results in the characteristic TMS-sound while providing no effective stimulation. During the post-training stimulation period, participants were asked to remain seated as still as possible with their eyes open. Coil positioning and orientation to stimulate M1 or dPMC were kept constant by using the neuro-navigation system. APB surface EMG was recorded across the post-training stimulation period to verify that the right hand was relaxed, to assess potential changes of APB MEP amplitude sizes across time in the M1 group and to demonstrate that post-training dPMC rTMS did not induce suprathreshold M1 stimulation caused by current spread due to the proximity of stimulation sites. To assess potential rTMS-induced modulation of corticospinal excitability (CSE), 20 APB-MEPs that were elicited at 0.1 Hz with the stimulator output set to an individually adjusted intensity to produce MEPs of ~ 1 mV were recorded before introduction of the task and onset of training (MEPpre) and immediately following the post-training rTMS intervention (MEPpost; Fig. 1).

### Data acquisition and analysis

Custom MatLab (Mathworks, Natick, USA) scripts were used to record the timing of key presses and to extract measures of speed performance and accuracy. Speed was defined as the average time (seconds) needed to execute correct sequences within a given block of task training (correct sequence duration, CSD). Accuracy was defined as the ratio of the number of correctly performed sequences per block divided by the maximum number of correct sequences per block (i.e., 12). To take potential interindividual differences into account with respect to the strategy to improve task performance (e.g., prioritize speed over accuracy, or vice versa), we additionally quantified task performance using a performance index (PI) that incorporates both equally important components of task performance^37^.

$$PIx=100*{e}^{-CSDx}*{e}^{ACCx-1}$$

where x = block of trials.

Effects of repeated practice on task performance across the initial training session and the delayed retest session (i.e., online performance changes) were assessed by applying separate repeated measures analyses of variance (rmANOVA) to the training and retest speed, accuracy, and combined PI values with *Group* (post-training M1, dPMC, SHAM rTMS) as the between-subject factor and *Block* (e.g., B1, …, B14) as within-subject factor. This allowed us to investigate potential between-group differences in terms of the magnitude and rate of online performance changes as a function of repeated practice within the training and delayed retest sessions. Offline consolidation effects were computed as the difference between the individual end-of-training baseline (EoT; i.e., average performance across the last 4 blocks of the training session) and the performance at the beginning of the delayed retest (BoRT, performance in the first block of the delayed retest) so that positive values indicate offline performance increments relative to EoT and negative values indicate offline performance impairments. We chose to use only the first block of retesting to compute offline consolidation to not confound the consolidation measure with additional online learning.

Electromyographic data was recorded using “CED Signal” (Cambridge Electronic Design Ltd., Cambridge, England) and manually processed with the “palMEP” tool (Perellón-Alfonso et al. 2018). All recorded MEPs were evaluated separately and discarded if any artefacts (pre-activation, voluntary movements etc.) were observed in the EMG recordings. For evaluation of potential rTMS-induced effects on CSE, we averaged MEP amplitude sizes across MEPpre and across MEPpost assessments and applied a rmANOVA with the within-subject factor *Time* (MEPpre, MEPpost) and the between-subject factor *Group* to these values. To assess alterations of CSE across the 20 min of the post-training 1 Hz rTMS intervention in the M1 group, MEP amplitude sizes were averaged across blocks of 4 min (240 frames) each and analysed using rmANOVA with the within-subject factor *Time* (minutes 1–4, 5–8, 9–12,13–16, 17–20).

Spearman´s rank correlation coefficient was applied to assess associations of rTMS-induced modulation of CSE (difference between MEPpost and MEPpre; MEPpost) and the magnitude of offline performance changes.

All statistical analyses were performed with SPSS 25 (SPSS, Chicago, IL, USA). For all statistical tests, the alpha level was set to p < 0.05. rmANOVAs were checked for violation of sphericity and degrees of freedom and p-values were corrected accordingly if necessary (Greenhouse–Geisser correction).

## Results

### Participant characteristics

Demographic information and characteristics of the three groups (M1, dPMC, SHAM rTMS) corresponding to the three different post-training rTMS interventions are detailed in Table 1. There were no significant between-group differences in terms of age, sex, depressive symptoms as assessed by the BDI, handedness as assessed with the EHI, the baseline resting motor threshold, nor the stimulation intensity required to elicit MEPs of approximately 1 mV (all p ≥ 0.114). There were also no significant differences in terms of vigilance before training (p = 0.161), and before the delayed retest (p = 0.265) as assessed with the Stanford-Sleepiness-Scale. This collectively indicates that potential between-group differences in terms of online and offline motor learning are unlikely to be generated by differences in group demographics and characteristics.

### Offline low-frequency rTMS-induced effects on corticospinal excitability

**Figure 2:**
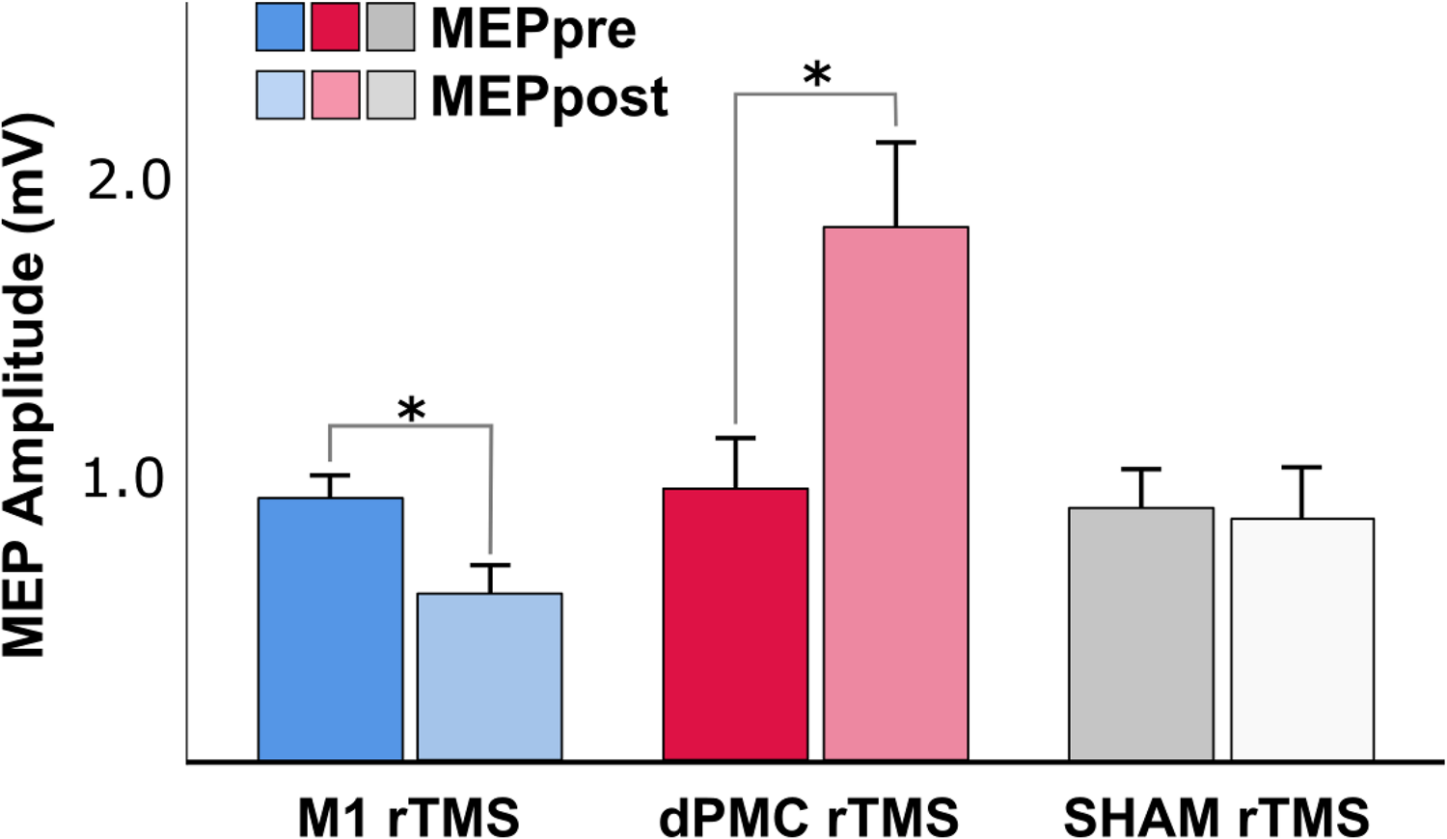
1 Hz rTMS-induced effects on corticospinal excitability. Mean amplitudes of 20 motor evoked potentials (MEPs) before the training session (MEPpre) and immediately after (MEPpost) 20 min of post-training 1 Hz rTMS of the primary motor cortex (M1 rTMS), the dorsal premotor cortex (dPMC rTMS), or sham rTMS. Error bars indicate the standard error of the mean. The asterisks indicate significant differences between the MEPpre and MEPpost assessments, p < 0.05.

Pre-training baseline CSE as indexed by average MEP amplitude was 0.91 mV (CI 0.73–1.09) in the M1 rTMS group, 0.93 mV (CI 0.57–1.28) in the dPMC rTMS group, and 0.88 mV (CI 0.59–1.16) in the SHAM rTMS group and, thus, did not significantly differ between groups (F_(2,45)_ = 0.037, p = 0.964). A rmANOVA including the between-subject factor *Group* (M1, dPMC, SHAM) and the within-subject factor *Time (MEPpre, MEPpost)* revealed a significant main effect of *Group (*F_(2,45)_ = 5.898, p = 0.005) and a significant *Time x Group* interaction (F_(2,45)_ = 7.684, p = 0.001) in the absence of a main effect of *Time* (F_(1,45)_ = 1.588, p = 0.214). Subsequent rmANOVA showed that the significant interaction of *Time* x *Group* was driven by a significant decrease of the average MEPpost amplitude compared to the average MEPpre amplitude in the M1 rTMS group (− 0.337 mV, CI − 0.566 to − 0.109, *Time:* F_(1,15)_ = 9.886, p = 0.007), while there was a significant MEP amplitude increase in the dPMC rTMS group (+ 0.868 mV, CI 0.202–1.535; *Time:* F_(1,15)_ = 7.706, p = 0.014), and no relevant change of CSE in the SHAM group (− 0.037 mV, CI − 0.486–0.412; *Time:* F_(1,15)_ = 0.031, p = 0.863; Fig. 2). Collectively, this demonstrates that post-training CSE was significantly and differentially modulated depending on the type/location of the post-training rTMS intervention. While, as expected, post-training 1 Hz M1 rTMS significantly decreased the size of the MEP amplitudes, post-training 1 Hz rTMS of the dPMC significantly increased CSE compared to the pre-training assessment. Evaluation of the evolution of CSE across the post-training 1 Hz rTMS intervention directed to M1 showed that the MEP amplitude size decreased from an average of 0.382 mV (CI 0.017–0.746) during the first four minutes to 0.245 mV (CI 0.088–0.402; rmANOVA *Time*: F_(1.15,16.13)_ = 1.558, p = 0.234) across the last four minutes of stimulation (please note that data of one participant were excluded from this ANOVA due to missing data at the beginning of the post-training rTMS stimulation period). No MEPs were detected during the post-training 1 Hz SHAM rTMS intervention or during the 1 Hz dPMC stimulation. The latter observation demonstrates absence of supra-threshold current spread despite the proximity of the dPMC stimulation site to M1.

### Behavioural results

#### Accuracy

**Figure 3:**
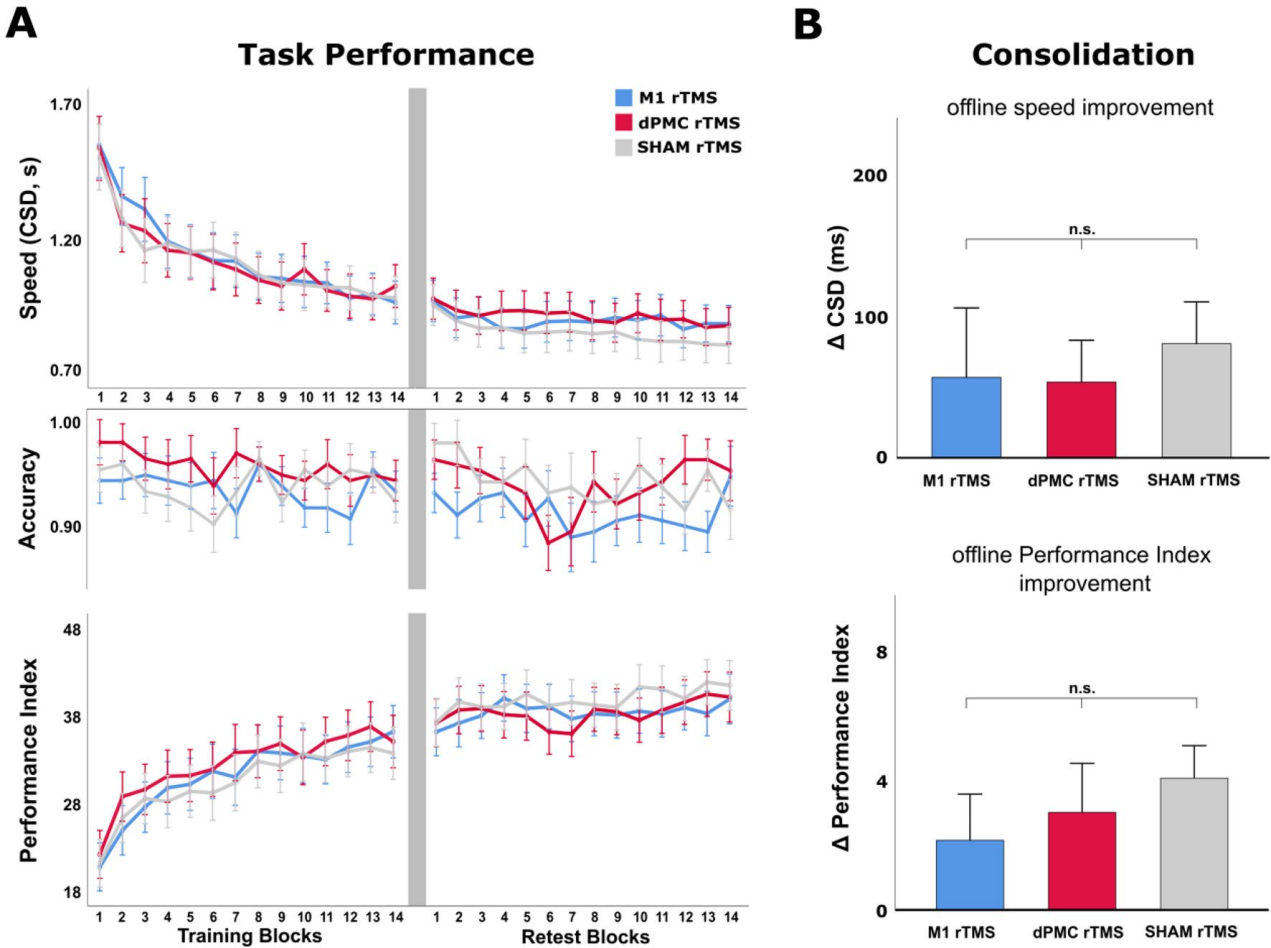
Behavioural results. (**A**) Performance changes of all three experimental groups (post-training M1 rTMS, dPMC rTMS, and SHAM rTMS) across blocks of task execution in terms of speed (mean duration of a correct sequence, CSD), accuracy (ratio of the number of correct sequences per block/number of maximum correct sequences per block), and the combined performance index. The grey column between the training and retest graphs represents the 8-h break interval. Error bars indicate the standard error of the mean. (**B**) Offline task performance changes (difference) between end-of-training (mean performance across the last 4 blocks of the training session) and beginning of the delayed retest (first block of delayed retesting). Note that differences were computed such that positive values indicate offline performance improvements (i.e., offline learning). Error bars indicate the standard error of the mean (n.s.: not significant).

Accuracy of task performance was very high amounting to 0.937 (CI 0.924–0.951) across the training and delayed retest sessions, indicating that participants, on average, produced less than one incorrect sequence per block (Fig. 3A). Moreover, a rmANOVA conducted on the accuracy measure revealed no significant effect for the factors *Group* (M1, dPMC, SHAM rTMS) and *Block* (B1, …, B14), or the interaction of both factors across the initial training session (all p ≥ 0.385) as well as across the delayed retest session (all p ≥ 0.117). Collectively, this indicates that motor sequence performance in terms of accuracy was not modulated by repeated task practice, potentially because participants performed at or close to accuracy ceiling even at the beginning of the experiment. Online task learning and potential effects of the rTMS intervention on offline consolidation were therefore assessed using speed as the primary performance measure.

#### Training session–online learning

A rmANOVA conducted on the speed performance measure (CSD) with the between-subject factor *Group* (M1, dPMC, SHAM rTMS) and the within-subject factor *Block* (B1, …, B14) revealed a significant main effect of *Block* (F_(3.79,170.35)_ = 54.745, p < 0.001) in the absence of a significant main effect of *Group* (F_(2,45)_ = 0.011, p = 0.989), or a significant interaction of both factors (F_(7.57,170.35)_ = 0.763, p = 0.629). Average baseline CSD in the first block of the training session amounted to 1.624 s (CI 1.480–1.769) and did not significantly differ between groups (F_(2,45)_ = 0.038, p = 0.963). Speed performance reached a similar asymptotic plateau in all groups at the end of the training session (EoT, average performance across the last 4 blocks of the training session; 1.068 s, CI 0.977–1.160), as indicated by a non-significant effect of *Block* (F_(2.45,110.45)_ = 1.669, p = 0.186) and *Group* (F_(2,45)_ = 0.003, p = 0.997), and the absence of a significant interaction of *Block* × *Group* (F_(4.91,110.45)_ = 1.164, p = 0.332). The above results, collectively, indicate that participants in all groups increased speed of performance at a similar rate across the initial training session and reached similar asymptotic performance at the end of the training session (EoT), against which consolidation effects were assessed. This rules out that potential effects of the following offline rTMS intervention on consolidation may have been confounded by differences of online skill acquisition during the training session.

#### Delayed retest—offline consolidation

Offline performance changes as measure of consolidation were assessed between EoT (average performance across the last 4 blocks of training) and the performance at the beginning of the delayed retest (BoRT, first block of delayed retesting). rmANOVA including the within-subject factor *Time* (EoT, BoRT) and the between-subject factor *Group* revealed a significant main effect of the factor *Time* (F_(1,45)_ = 8.605, p = 0.005), which was driven by an offline speed (CSD) improvement between EoT and BoRT of 63.8 ms (CI 20.9–106.6) across all groups. There was no significant main effect of the factor *Group* (F_(2,45)_ = 0.005, p = 0.995), and, most importantly, no significant interaction of *Group* x *Time* (F_(2,45)_ = 0.162, p = 0.851; Fig. 3B). This indicates that, although 1 Hz rTMS of M1 and 1 Hz rTMS of dPMC significantly modulated post-training CSE in different directions, neither type of intervention had a relevant effect on the consolidation process compared with the sham intervention. Of note, we had decided to use the first block of retesting to compute offline performance changes against EoT to not confound the consolidation measure with additional task training and potential online learning effects. However, the above results remain similar if averages of different subsets of delayed retest blocks (e.g., average of the first two, three, or four retest blocks) were used to compute the consolidation measure.

We further assessed whether the post-training rTMS intervention may have affected potential differences in online learning across the delayed retest session by applying a rmANOVA on the CSD values across the 14 blocks of delayed retesting. This analysis revealed a significant main effect of *Block* (F_(6.37,286.74)_ = 7.700, p < 0.001), while there was no significant main effect of *Group* (F_(2,45)_ = 0.209, p = 0.812), nor a significant interaction of *Group* x *Block* (F_(12.74, 286.74)_ = 1.155, p = 0.314), indicating similar online learning across the delayed retest session independent of the previous post-training rTMS intervention. In addition to the observation of significant offline performance improvements from EoT to BoRT across all groups, the significant Block effect in this rmANOVA suggests that the lack of a between-group difference in offline consolidation cannot be attributed to reaching a ceiling in performance speed at the end of the initial training session. In accordance with the above results, non-parametric correlation analyses revealed no relevant association of the magnitude of offline performance changes between EoT and BoRT with the amplitude of the MEPpost amplitudes (r = 0.139, p = 0.346) nor with the magnitude of rTMS-induced changes in CSE (Delta MEPpost—MEPpre; r = − 0.055, p = 0.713).

#### Performance index

Although average accuracy across blocks and groups was not relevantly modulated by the task, we chose to additionally apply a performance index incorporating speed and accuracy measures to account for potential interindividual differences with respect to the strategy to improve task performance (e.g., prioritize speed performance at the expense of accuracy). A rmANOVA conducted on the PI values across the training session revealed a significant main effect of *Block* (F_(6.08,272.77_ = 41.049, p < 0.001) in the absence of a significant effect of *Group* and absence of a significant interaction of *Group x Block* (both p ≥ 0.837), indicating similar online learning across the training session (Fig. 3A) Initial average PI of the first block of training amounted to 21.38 (CI 18.43–24.33) and reached an average of 34.04 (CI 31.08–36.99) across the last four blocks of training (EoT). Similar to the speed performance analysis, a rmANOVA with the within-subject factor *Time* (EoT, BoRT) and the between-subject factor *Group* was conducted to assess potential differences in offline consolidation. While there was a significant effect of the main factor *Time* (F_(1,45)_ = 14.948, p < 0.001), this analysis revealed no significant effect of *Group* nor a significant interaction of factors *Time* x *Group* (both p ≥ 0.483). The significant main effect of time was driven by an offline average PI improvement between EoT and BoRT of 2.81 (CI 1.36–4.27) across all groups (Fig. 3B), indicating significant offline learning during the consolidation period irrespective of the type of post-training rTMS intervention (again, results were similar when different subsets of retest blocks were defined as BoRT). Performance increased further across the 14 delayed retest session blocks in all groups as indicated by a significant effect of *Block* (F_(8.14, 366.37)_ = 2.665, p = 0.007), while there was no significant main effect of *Group* or a significant interaction of *Block* x *Group* (both p ≥ 0.625), demonstrating that also online learning during delayed retesting was not modulated by any type of the prior post-training rTMS intervention. Moreover, there were no significant correlations of the magnitude of offline performance changes between EoT and BoRT with the amplitude of the MEPpost amplitudes (r = 0.071, p = 0.630) or the magnitude of rTMS-induced changes in CSE (Delta MEPpost—MEPpre; r = -0.044, p = 0.766). Consistent with the analyses of movement speed above, these results collectively indicate that the stimulation intervention had no impact on motor sequence performance as assessed with a speed-accuracy aggregate measure.

## Discussion

The current study was primarily designed to investigate the function of the premotor cortex and its potential interaction with the primary motor cortex during early post-training motor memory consolidation. Our main findings were that targeting the dPMC with “inhibitory” low-frequency rTMS immediately after explicit motor sequence training enhanced the excitability of M1 output neurons but had no modulatory effect on subsequent offline skill consolidation. As expected, post-training 1 Hz rTMS applied to M1 induced a significant decrease of post-training CSE. However, also post-training 1 Hz rTMS of M1 failed to induce a relevant behavioural effect on subsequent motor memory consolidation. Importantly, online learning across the initial training session was similar among all three experimental groups (i.e., post-training sham rTMS, dPMC rTMS, and M1 rTMS) with respect to all assessed measures of performance (i.e., speed, accuracy, and the combined performance index). This rules out that potential post-training rTMS-induced effects on consolidation or CSE were confounded by differences of online skill acquisition within the initial training session. Moreover, all three experimental groups demonstrated similar significant between-session performance increments (i.e., offline-learning) independent of the level of post-stimulation CSE or changes in CSE between the pre-training baseline and the post-stimulation assessments. Collectively, the present findings challenge the idea that any potential role that the dPMC or M1 might have in early consolidation can be disrupted by post-training low-frequency rTMS. Furthermore, although our findings do not exclude a role of dPMC or M1 in early consolidation, it is highly unlikely that this role is mediated by or apparent as changes in CSE.

Previous studies that investigated the role of the premotor cortex in motor learning reported that the post-training consolidation process may be modulated by NIBS of the dPMC, indicating its relevant involvement in motor memory consolidation. However, results of these studies are inconsistent in terms of the direction of effects on motor memory consolidation induced by the application of either “excitatory” or “inhibitory” NIBS protocols. While Boyd and co-workers^23^ reported a facilitation of motor memory consolidation by application of “excitatory” 5 Hz rTMS to the dPMC (and no effect of 1 Hz rTMS), Kantak and co-workers^25^ demonstrated impaired motor consolidation when the dPMC was targeted with “excitatory” anodal tDCS. On the other hand, application of “inhibitory” cathodal tDCS to the dPMC resulted in impaired delayed reproduction of a motor sequence in one study^27^, while the same intervention was reported to facilitate post-training stabilization of a newly learned motor sequence in another study^26^. Moreover, the interpretation of these findings in terms of a role of the dPMC specifically during early offline motor-memory consolidation is difficult because in the above studies NIBS was applied either before^23,26,27^, or during^25^ task training. Therefore, it cannot be excluded that the reported effects on subsequent consolidation were confounded by an interaction of NIBS-induced effects on dPMC (or persisting after-effects if applied before training) with processing of online skill acquisition during training. However, studies in which NIBS of dPMC was applied after motor sequence training—thus exclusively targeting the direct interaction of NIBS-induced modulation of dPMC with early post-training offline motor memory processing—also produced mixed results. While Meehan and co-workers^24^ reported facilitation of motor sequence consolidation by immediate post-training “inhibitory” 1 Hz rTMS of the dPMC, which might suggest an inhibitory influence of the dPMC during early consolidation, Nitsche and co-workers^28^ demonstrated enhanced consolidation by delayed post-training “excitatory” anodal tDCS of the dPMC during sleep. However, immediate post-training “excitatory” anodal tDCS of the dPMC exerted no effect on motor sequence consolidation in a group of healthy older people in a recent study from our group^38^. Findings of the latter study are in line with the current results that do not provide evidence of a relevant involvement of the dPMC during early post-training consolidation following explicit training-induced sequential motor skill acquisition in young adults. This interpretation may be supported by a recent meta-analysis that suggests that motor sequence learning is mainly and particularly driven by contributions of the basal ganglia, whereas the premotor cortex as well as the cerebellum play a subordinate or negligible role for learning-specific aspects in motor sequence learning^39^. Collectively, if the above body of evidence indeed reflects absence of the dPMC in motor sequence learning, then this interpretation may perhaps explain the inconsistent results of the dPMC NIBS studies.

Another potential explanation may be that the diverging results of the dPMC NIBS studies are driven by different task characteristics in terms of explicit or implicit motor sequence skill acquisition. Explicit and implicit sequence learning are believed to be sustained by at least partly different neural substrates and involvement of the premotor cortex was reported to be particularly critical in explicit sequence learning^22,29,30^. However, in the current study as well as in our previous study^38^, which both indicated no relevant role of the dPMC during early consolidation, we used a purely explicit motor sequence learning task. While this does not exclude a role of the dPMC during explicit online learning, it still questions a relevant role of the dPMC at least during early consolidation of explicitly acquired sequential motor skills. Moreover, findings in terms of involvement of the dPMC during consolidation were also inconsistent among the studies that applied implicit serial reaction time tasks^24–26,28^. Collectively, this body of evidence does not support the conclusion that involvement of the dPMC during post-training consolidation is determined by whether motor sequence skill was acquired under explicit or implicit conditions.

As expected, and consistent with earlier research^40–42^, 1 Hz rTMS of M1 decreased CSE across the post-training intervention, resulting in significant CSE suppression in the post-stimulation (MEPpost) assessment. Interestingly, post-training 1 Hz rTMS of the dPMC induced the opposite effect on CSE, i.e., significant facilitation of post-training CSE. Besides the fact that we did not detect any MEPs during the post-training dPMC rTMS intervention, this excludes suprathreshold current spread from the dPMC stimulation to M1 which would likely have resulted in decreased CSE. One might then still ask whether facilitation of CSE by 1 Hz rTMS of the dPMC may be caused by subthreshold current spread to M1. However, we think that this is unlikely as we are not aware of any studies that suggest facilitation of CSE by subthreshold low-frequency M1 rTMS. Thus, facilitation of CSE by post-training 1 Hz rTMS of dPMC rather indicates modulation of M1 output neuron excitability via dPMC > M1 projections. This interpretation may be supported by previous studies that also demonstrated modulation (albeit depression) of CSE by low-frequency rTMS^18,43^ or continuous theta burst stimulation^16,17^ of the dPMC in the absence of a prior motor training intervention. Facilitation of M1 output neuron excitability by post-training “inhibitory” 1 Hz rTMS of dPMC in the current study points to the possibility of a tonic inhibitory input of the dPMC on M1 during early consolidation after explicit sequence skill acquisition that reflects a state-dependent interconnection of M1 and dPMC. This view is, however, challenged by findings of Meehan and co-workers^24^ who did not report relevant changes of CSE following post-training low-frequency rTMS of dPMC after implicit motor sequence learning.

Another interesting aspect of our findings was that also targeting M1 with low-frequency rTMS after motor sequence training did not affect consolidation despite rTMS-induced reduction of post-training CSE. This was unexpected given previous findings suggesting that immediate post-training inhibition of M1 by 1 Hz rTMS interventions was shown to not only block offline consolidation of simple ballistic finger movement skills^3^, but to also block over-the-day consolidation of more complex implicitly-acquired finger tapping sequences in a study by Robertson and co-workers^4^. The induction of consolidation during wakefulness over the day was, furthermore, linked to post-training CSE by Tunovic and colleagues^8^ who reported that acquisition of a sequential motor skill under explicit conditions induced an immediate post-training decrease of CSE which was associated with absence of offline-learning, while performing the same task under implicit conditions did not induce a post-training CSE decrease and was followed by significant offline performance gains. Moreover, the magnitude of offline-performance increments was shown to be associated with the level of immediate post-training CSE. Interestingly, the induction of offline performance gains subsequent to explicit task training was restored when the immediate post-training CSE decrease was prevented by remote rTMS^8^. Facilitation of consolidation following training-induced acquisition of sequential motor skills by post-training application of anodal transcranial direct current stimulation (tDCS) of M1^38,44,45^ may, thus, be explained by a similar mechanism, i.e., facilitation of immediate post-training CSE. Collectively, these observations have been suggested to indicate that the level of immediate post-training CSE represents a neurophysiological signal that determines whether and how much training-induced motor representations are promoted offline during wakefulness. However, results with respect to facilitation of consolidation by offline application of tDCS to M1 are inconsistent for both explicit^46^ and implicit motor sequence learning^47^. Moreover, we did not detect an immediate post-training decrease of CSE as described by Tunovic and co-workers^8^, although a purely explicit motor sequence learning task was applied in the current study. Moreover, neither subsequent post-training depression of CSE induced by 1 Hz rTMS of M1 nor significant remote facilitation of post-training CSE induced by 1 Hz rTMS of dPMC influenced the magnitude of offline skill performance changes between sessions. This suggests that neither (i) dichotomic differential modulation of immediate post-training CSE by prior implicit or explicit sequential motor skill acquisition nor (ii) determination of induction of consolidation by the level of post-training CSE are generalizable principles in motor sequence learning.

Of note, considering the evidence for a distinct role of spinal cord plasticity in motor sequence learning (e.g.,^15^), we would like to point to a limitation of our study that we have no information on if and to which extent spinal cord plasticity was modulated or contributed to the current findings.

In conclusion, despite evidence of an interaction of dPMC and M1 at the level of CSE, current findings indicate that the post-training consolidation process following motor sequence skill acquisition under explicit conditions is not accessible to perturbation of dPMC or M1 by low-frequency rTMS. In contrast to previous findings that suggest modulation of motor memory consolidation by post-training NIBS of dPMC^24^ and M1^4^ in implicit motor sequence learning, this may suggest that offline processing of explicitly acquired motor sequence skills is a rather robust process that is at least not malleable by low-frequency rTMS-induced manipulation of just single nodes of the motor learning network. Our results further indicate that offline processing of explicitly acquired motor sequences is neither mediated nor reflected by post-training changes in CSE which questions the generalizability of this marker as a predictor of consolidation beyond specific learning tasks.

## Data Availability

The data used to support the findings of this study are available from the corresponding author upon reasonable request.
